# Supplementary material for: Gut dysbiosis contributes to the development of Budd-Chiari syndrome through immune imbalance
Source: mSystems. 2024 Aug 21;9(9):e00794-24. doi: 10.1128/msystems.00794-24 (PMC11406926; doi:10.1128/msystems.00794-24)
Supplement: Supplemental figures — Figures S1 to S4. [file msystems.00794-24-s0001.pdf]

Supplementary Figure Legends

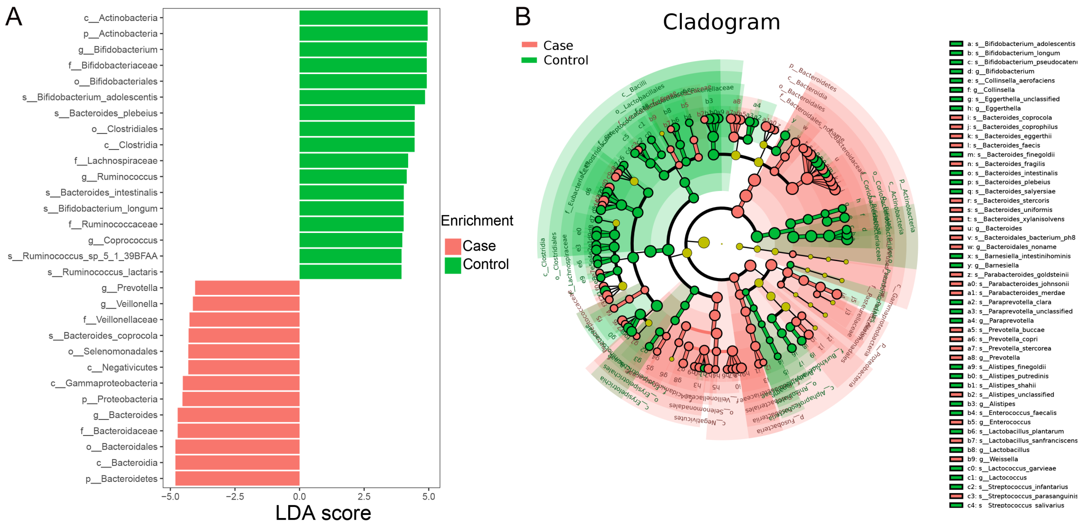

Supplementary Figure 1 Comparative analysis of metagenomics between healthy populations and patients with B-CS. (A) Linear discriminant analysis (LDA) effect size identified the most differentially abundant taxa between the two groups. B-CS enriched taxa are indicated with a positive LDA score, and taxa enriched in controls have a negative score. Only taxa meeting an LDA significant threshold of >2 are shown. (B) Differential species evolutionary cladogram, circles radiating from inside to outside represent phylum to species taxonomic levels. Each small circle at a different taxonomic level represents a taxon at that level, and the small circle diameter size is proportional to the relative abundance size.

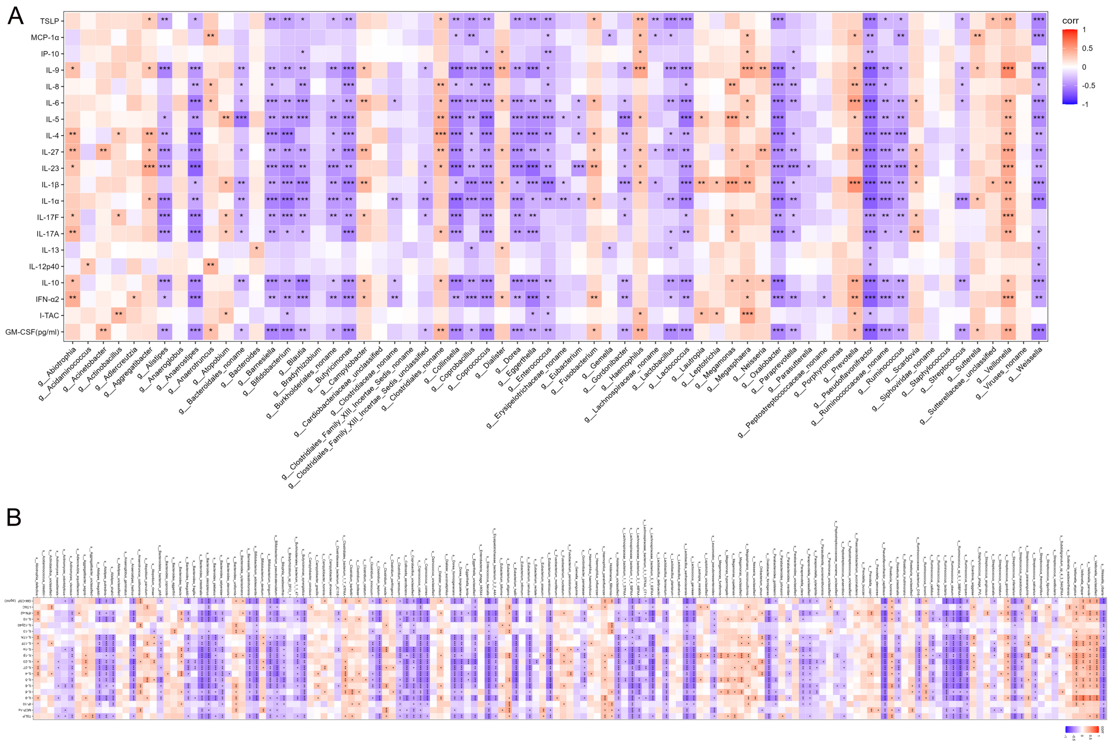

Supplementary Figure 2 The association of Cytokine disorder and gut dysbiosis in B-CS. (A) The heat map showed multiple dysregulated cytokines were positively associated with significantly elevated microbiota in the B-CS group, while negatively associated with significantly reduced microbiota at the genus level. (B) The heatmap showed association analysis at the species level. * P < 0.05, **P < 0.01, ***P < 0.001

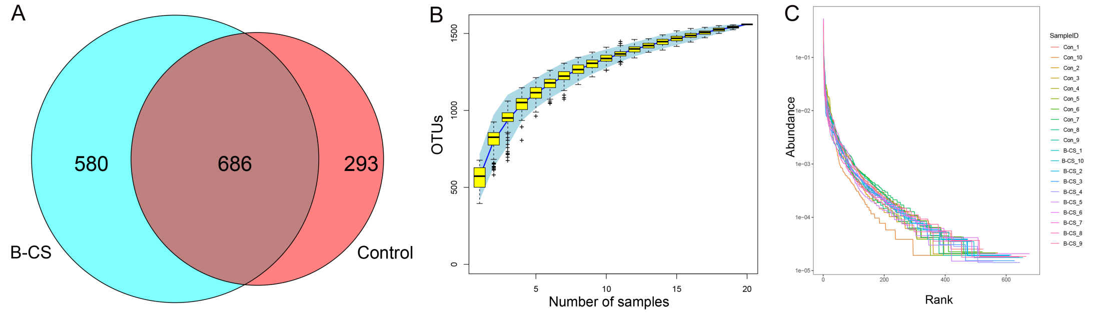

Supplementary Figure 3 Quality control of sequencing data from B-CS-FMT mice and control. (A) Common and unique OTUs between different sample groups (n = 20) were analyzed and plotted as a Venn diagram based on OTUs cluster analysis results. (B) Box plot locations of species accumulation curves tend to flatten out explained that species in this environment did not increase significantly with sample size. (C) The rank abundance curves showed that species abundance and evenness can be used for further

analysis.

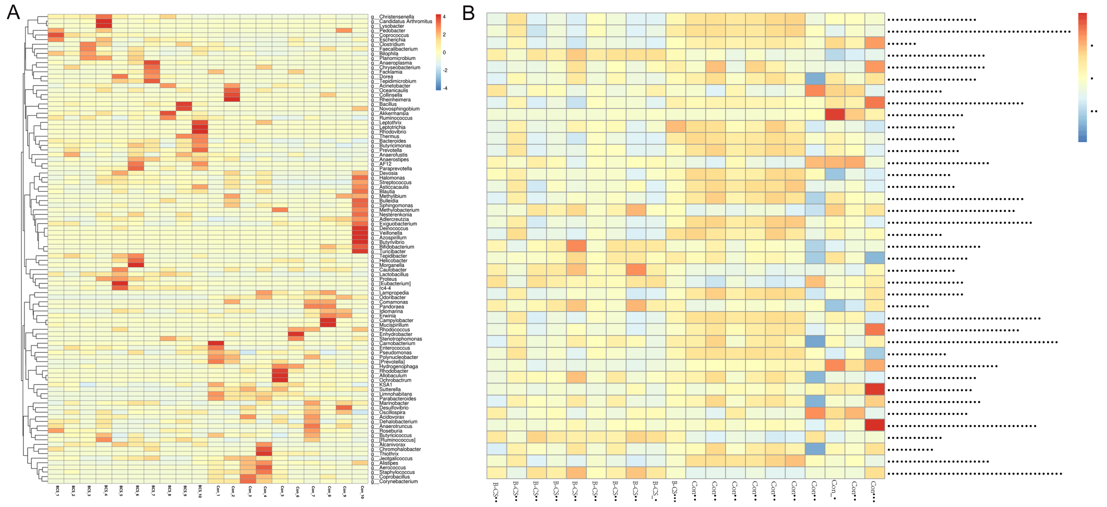

Supplementary Figure 4 Heatmap of differential gut microbiota and differential function prediction between B-CS-FMT mice and control. (A) Heatmap of the 100 most abundant bacterial genera in control and B-CS-FMT mice. The different bacteria genera were identified by clustering analysis. The color represents the relative abundance of each bacterial genus and the changes in color from blue to red indicates a transition from low to high abundance. (B) The heatmap represents the functional abundance value by a defined color depth using the Kyoto Encyclopedia of Genes and Genomes (KEGG) database. Different colors represent different functional entries, corresponding to the right legend; the horizontal axis represents different samples or groups, and the vertical axis represents the relative abundance of each functional entry.
